# Limb development in skeletally-immature large-sized dogs: A radiographic study

**DOI:** 10.1371/journal.pone.0254788

**Published:** 2021-07-23

**Authors:** Mariana Roccaro, Alessia Diana, Nikolina Linta, Riccardo Rinnovati, Marzia Freo, Angelo Peli

**Affiliations:** 1 Department of Veterinary Medical Sciences, *Alma Mater Studiorum* University of Bologna, Bologna, Italy; 2 Department of Statistical Sciences, *Alma Mater Studiorum* University of Bologna, Bologna, Italy; University of Lincoln, UNITED KINGDOM

## Abstract

Despite the extreme morphological variability of the canine species, data on limb development are limited and the time windows for the appearance of the limb ossification centres (OCs) reported in veterinary textbooks, considered universally valid for all dogs, are based on dated studies. The aim of this study was to acquire up-to-date information regarding the arm, forearm and leg bone development in skeletally-immature large-sized dogs from 6 weeks to 16 weeks of age. Nine litters of 5 large-sized breeds (Boxer, German Shepherd, Labrador Retriever, Saarloos Wolfdog, White Swiss Shepherd Dog) were included, for a total of 54 dogs, which were subject to radiographic examination on a bi-weekly basis. The appearance of 18 limb OCs was recorded and 14 radiographic measurements were performed; their relationship with age and body weight was investigated and any breed differences were analysed using different statistical non-parametric tests. The number of OCs present was significantly different at 6 and 8 weeks of age between the investigated breeds. The appearance of the OCs occurred earlier in the Saarloos Wolfdog, while the Labrador Retriever was the later breed. In Boxers and Labrador Retrievers, various OCs showed a delayed appearance compared to the data reported in the literature. The number of OCs was strongly and positively correlated to body weight. Breed differences were also observed in the relative increase of the measured OCs and were not limited to dogs of different morphotypes. Statistically significant differences were most frequently observed between Saarloos Wolfdogs and the other breeds. The OCs that showed a greater variability in their development were the olecranon tuber, the patella and the tibial tuberosity. Their increase was more strongly correlated with the dog’s age and body weight. Our data strongly suggest that differences in limb development exist in dog breeds of similar size and morphotype.

## Introduction

Dogs are the most morphologically variable domesticated mammals, displaying extreme variations in terms of size, shape and body weight. Breed-specific differences in growth patterns have been observed, with toy, small and medium-sized dog breeds reaching their adult weight at around 9–10 months of age, whereas large-sized dog breeds reach their adult weight at around 15 months. Furthermore, additional differences in growth patterns appear to exist in breeds of similar size [1].

Knowledge of normal bone development processes is essential in the clinical setting to distinguish normal from pathological conditions and to estimate the age of growing dogs. Skeletal development can be assessed through the radiographic evaluation of the appearance and fusion of the limb bone ossification centres (OCs). The existing literature concerning the normal development of the limb OCs in dogs has been recently reviewed [2]. Most of the published research papers date back to the 1950s-1960s [3–15] and despite the amount of information provided, these studies have many limitations: they often include a small sample of dogs, usually less than 20 animals with dogs actually radiographed at each time point being even less; the observation protocols are not always specified; the timeframe measurements and the anatomical landmarks are not homogeneous and, therefore, difficult to compare. Medium- and large-sized breeds like German Shepherd, Greyhound and Beagle are the most studied.

Notwithstanding these shortcomings, the timing of OC appearance and closure reported in various veterinary textbooks is mostly based on the aforementioned studies, although sometimes data do not correspond to the original reference or the source of information is not specified [16–25].

The aim of this study was to acquire up-to-date information regarding limb bone development in skeletally-immature large-sized dogs from 6 weeks to 16 weeks of age, based on measurements performed on the same subjects over time on a bi-weekly basis. More specifically, the radiographic appearance of the limb secondary OCs was recorded and radiographic measurements of long bone diaphyseal lengths and OC areas were performed. The relationship between age, body weight, number of OCs present and increase in the radiographic measurements of the diaphyseal lengths and OC areas was also investigated.

## Materials and methods

The protocol was approved by the animal-welfare body of the Istituto Zooprofilattico Sperimentale della Lombardia e dell’Emilia Romagna “Bruno Ubertini” (Protocol Number 7390 of 16/03/2017).

The dogs were all owned by private breeders, who were recruited according to their geographical proximity and availability. After a first contact via phone call, a meeting with the breeder was arranged in order to clearly explain the aims and procedures of the research and to acquire their informed consent signature.

### Animals

Nine litters of 5 different large-sized breeds were included, for a total of 54 dogs, 18 males and 36 females: Boxer (n = 10), German Shepherd (n = 7), Labrador Retriever (n = 15), Saarloos Wolfdog (n = 12) and White Swiss Shepherd Dog (n = 10).

All dogs were born by healthy bitches with normal gestation, parturition and post-partum course, regularly dewormed and vaccinated before mating. All the bitches were fed with pregnancy-specific commercial food. The dogs’ weight and clinical status were checked in order to eventually exclude non-healthy animals.

### Radiographic examination

Each dog was subject to radiographic examination on a bi-weekly basis from 6 weeks to 16 weeks of age, when not previously sold.

In order to minimize the exposure of dogs and personnel to X-rays, only the medio-lateral view of the right forelimb and hindlimb was obtained in two different radiographs with the beam centred on the structures of interest. Each dog was placed in right lateral recumbency with the limbs leaning on the plate. The focal distance was maintained constant (100 cm). To reduce any superimposition of structures, the right forelimb was extended cranially and ventrally to the sternum, the opposite limb was pulled in a caudo-dorsal direction and the neck was extended dorsally; the right hindlimb was extended ventrally and the opposite limb was drawn caudally. Care was taken not to over-rotate the limb and to avoid the overlapping of anatomical structures. The correct positioning was obtained using sandbags and gauze ties on the extremities of the limbs, without using pharmacological sedation.

Radiographic images were acquired with the portable X-ray unit Orange 1040HF (AcuMed Imaging Ltd.) assembled with the CR system iCR3600 (iCRco) or the Vita Flex (Carestream). The X-ray machine was set on 2.5 mAs, while kilovoltage was determined following Sante’s rule (2 x thickness + 40), according to the dog’s size and age [26].

**Fig 1:**
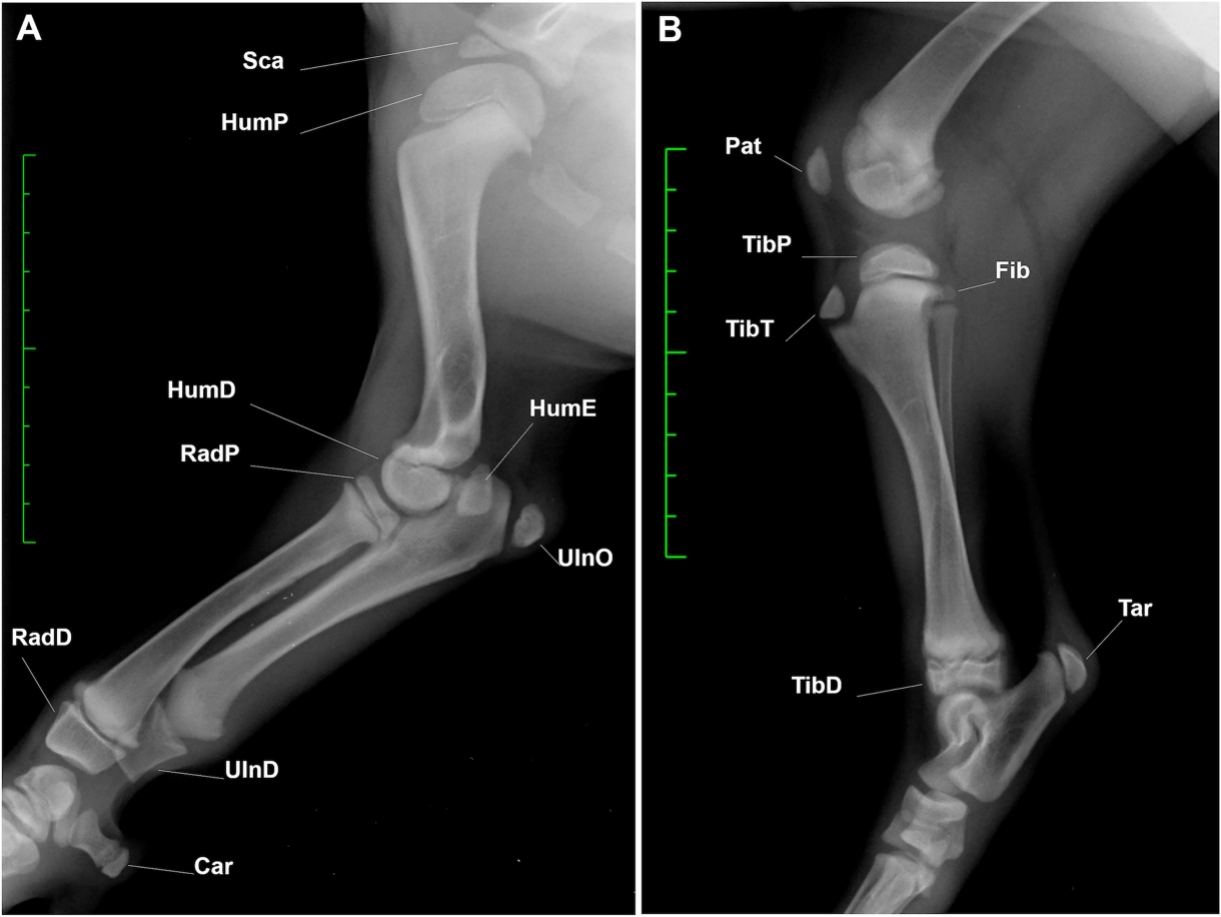
Radiographic appearance of the examined ossification centres. **A.** Saarloos Wolfdog, F, 6 weeks; medio-lateral view of the right forelimb; **B.** Saarloos Wolfdog, F, 8 weeks; medio-lateral view of the right hindlimb. Green scale bar: Distance between two hyphens is of 1 cm. Abbreviations: Sca = supraglenoid tubercule; HumP = proximal epiphysis of the humerus; HumE = epiphysis of medial epicondyle of the humerus; HumD = distal epiphysis of the humerus; RadP = proximal epiphysis of the radius; RadD = distal epiphysis of the radius; UlnO = olecranon tuber; UlnD = distal epiphysis of the ulna; Car = accessory carpal bone; Fem = distal epiphysis of the femur; Pat = patella; Fab = Fabellae; Pop = Popliteal bones; Fib = proximal epiphysis of the fibula; TibP = condyles of the proximal epiphysis of the tibia; TibT = tibial tuberosity; TibD = distal epiphysis of the tibia; Tar = calcaneal tuber.

For each dog and at each time point, the forelimb and hindlimb ossification centres (OCs) were evaluated on medio-lateral view by two operators with different levels of experience: a veterinary radiologist (Observer 1 –NL) and a final-year diagnostic imaging PhD student (Observer 2 –MR). Each OC was scored as either not present (stage 0) or present (stage 1). The OC was identified as a radiopaque area at the level of the future corresponding bone. A total of 18 OCs were examined: the supraglenoid tubercule (Sca), the proximal (HumP) and distal (HumD) epiphysis of the humerus, the epiphysis of medial epicondyle of the humerus (HumE), the proximal (RadP) and distal (RadD) epiphysis of the radius, the olecranon tuber (UlnO) and the distal epiphysis of the ulna (UlnD), the accessory carpal bone (Car), the distal epiphysis of the femur (Fem), the patella (Pat), Fabellae (Fab) and Popliteal (Pop) bones, the condyles (TibP) and tuberosity (TibT) of the proximal epiphysis of the tibia, the distal epiphysis of the tibia (TibD), the proximal epiphysis of the fibula (Fib) and the calcaneal tuber (Tar) (Fig 1).

Morphometry was also assessed by radiographic measurements performed by Observer 1 for all time points. On a sample of 10% of dogs at three time points (8, 10 and 14 weeks of age) radiographic measurements were repeated two times by Observer 2.

OsiriX Lite (Version 11, Pixmeo SARL, 2019) was used to process DICOM images, while Digimizer (Version 5.3.5; MedCalc Software bv, 2019) was used to analyse TIFF images.

**Fig 2:**
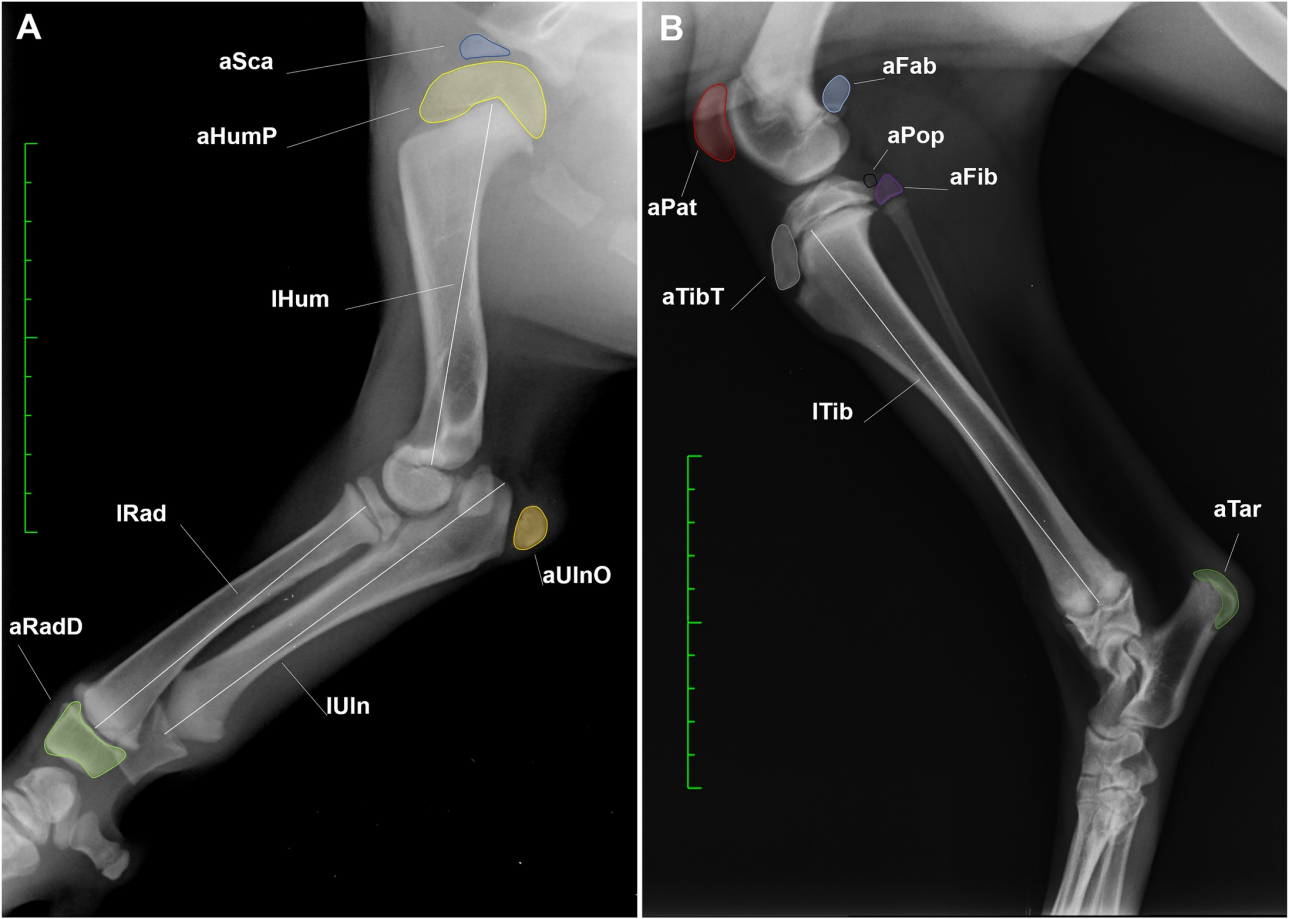
Radiographic measurements. **A.** White Swiss Shepherd Dog, F, 10 weeks, medio-lateral view of the right forelimb; **B.** Saarloos Wolfdog, F, 16 weeks, medio-lateral view of the right hindlimb. Diaphyseal length measurements (white lines) were performed by drawing a perpendicular line to the distal diaphyseal end of the bone and measuring the ossified diaphysis at its maximum length. The OC areas (outlined by the different coloured lines) were measured by drawing freehand the outline of the corresponding radiopaque area. Green scale bar: Distance between two hyphen is of 1 cm. Abbreviations: aSca = area of the supraglenoid tubercule; aHumP = area of the proximal epiphysis of the humerus; aUlnO = area of the olecranon tuber; aRadD = area of the distal epiphysis of the radius; lHum = diaphyseal length of the humerus; lRad = diaphyseal length of the radius; lUln = diaphyseal length of the ulna; lTib = diaphyseal length of the tibia; aPat = area of the patella; aFab = area of the fabellae; aPop = area of the popliteal bones; aFib = area of the proximal epiphysis of the fibula; aTibT = area of the tibial tuberosity; aTar = area of the calcaneal tuber.

Measurements were performed only on properly positioned radiographs. Diaphyseal length measurements were performed by using the “perpendicular lines” tool of the software. A line parallel to the metaphysis was drawn at its distal end; a perpendicular to this line was then automatically created and the diaphyseal length was measured at its maximum extent along this line. The start and end points of the diaphyseal length markers were represented by the radiotransparent space corresponding to the growth plate where the perpendicular line was positioned. The OC areas were measured by drawing freehand the outline of the corresponding radiopaque area. The following radiographic measurements were performed: area of the supraglenoid tubercule (aSca), area of the proximal epiphysis of the humerus (aHumP), diaphyseal length of the humerus (lHum), diaphyseal length of the radius (lRad), area of the distal epiphysis of the radius (aRadD), diaphyseal length of the ulna (lUln), area of the olecranon tuber (aUlnO), area of the patella (aPat), Fabellae (aFab) and Popliteal (aPop) bones, diaphyseal length of the tibia (lTib), area of the tibial tuberosity (aTibT), area of the proximal epiphysis of the fibula (aFib) and of the calcaneal tuber (aTar) (Fig 2).

### Statistical analysis

Statistical analysis was performed using Stata Statistical Software (Release 15; StataCorp, 2017).

Since data were analysed by breed and time, the number of subjects available at this level of detail was limited and we therefore recurred to non-parametric tests [27].

In order to manage missing data and at the same time minimise data loss, an available-case analysis (pairwise deletion) was performed. A p-value <0.05 was considered significant.

An “OC score” was calculated by summing the number of OCs present for each breed at each time point. Dogs with missing data on at least one OC were excluded from the “OC score” analysis. The score was calculated on a total of 14 OCs, with the exclusion of the supraglenoid tubercule and the proximal epiphysis of the fibula, which were often not viewable, and sesamoid bones fabellae and popliteal, which appeared at a much later stage.

To test for the presence of any significant differences in the number of OCs present at each time point between the investigated breeds, we used the Kruskal-Wallis one-way ANOVA. If the result of the test was significant, we used the Wilcoxon rank-sum test to determine which pairs of breeds differed. We applied the Bonferroni correction to adjust for multiple comparisons.

As regards the morphometric measures, we tested for the presence of significant differences in the relative increase of each measure between the investigated breeds by using the Wilcoxon rank-sum test; to test for the presence of significant differences in the absolute increase of each measure between the different time points within each breed we used the one-tailed Wilcoxon signed-rank test, which accounts for both sign and magnitude of the difference. For what concerned area measurements, the increase was calculated on the square root. We established three as the minimum number of measurements to perform the test.

Lastly, the presence of correlations (whether linear or not) between dog age, weight and the OC score or the relative increase of each radiographic measure was investigated by calculating the Spearman’s rank correlation coefficient. Correlations were investigated considering the sample population as a whole.

Intraclass correlation coefficients (ICCs) were calculated to evaluate intra- and inter- observer agreement. ICC values were categorized as poor (<0.50), fair (0.50–0.70), good (0.70–0.90) and excellent (>0.90) [28]. Values equal to zero and cases with less than 4 measurements available were excluded. In total, 258 measures were analysed. A p-value <0.05 was considered significant. Bland-Altman plots were constructed to display the intra- and inter- operator agreement for OC area and diaphyseal length measurements.

## Results

The dogs were in good health and made a steady weight gain through the observation period. The mean weight and standard deviation for each breed are reported in S1 Table.

**Fig 3:**
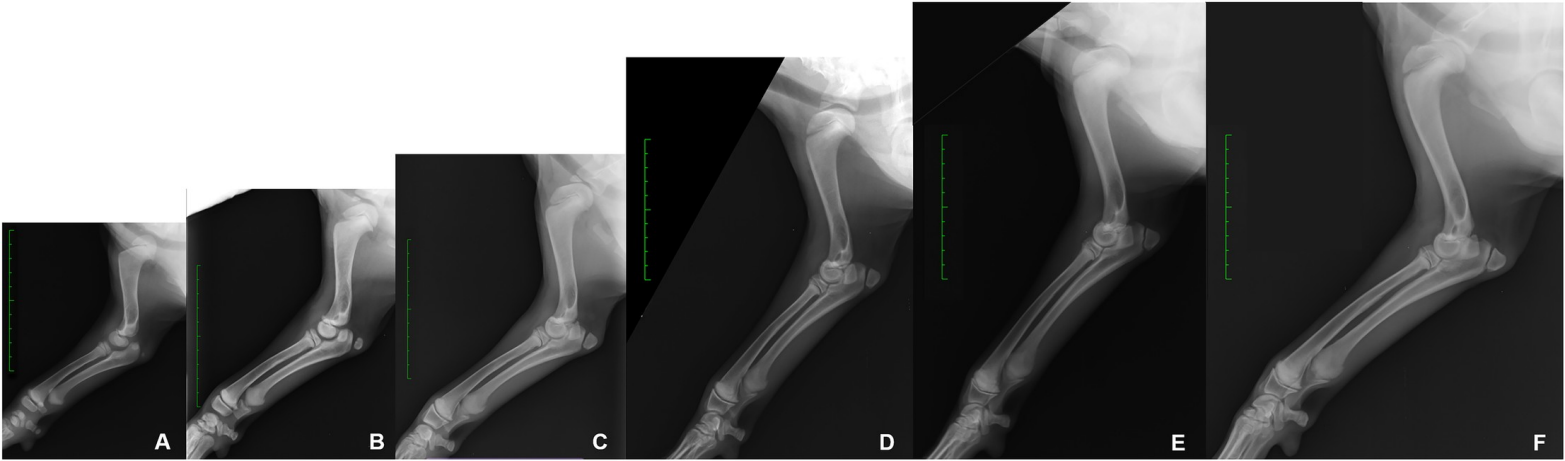
Representative series of radiographic images of the forelimb skeletal development. Saarloos Wolfdog, medio-lateral view of the right forelimb. A) 6 weeks of age; B) 8 weeks of age; C) 10 weeks of age; D) 12 weeks of age; E) 14 weeks of age; F) 16 weeks of age. Green scale bar: Distance between two hyphen is of 1 cm.

**Fig 4:**
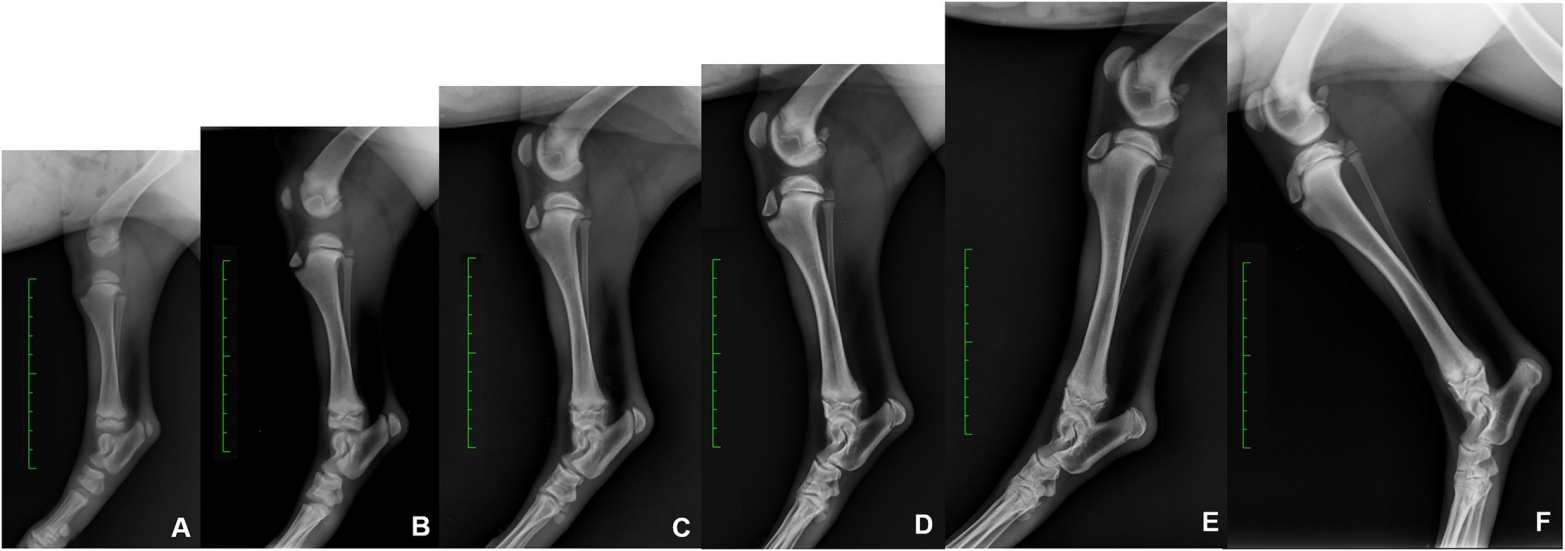
Representative series of radiographic images of the hindlimb skeletal development. Saarloos Wolfdog, medio-lateral view of the right hindlimb. A) 6 weeks of age; B) 8 weeks of age; C) 10 weeks of age; D) 12 weeks of age; E) 14 weeks of age; F) 16 weeks of age. Green scale bar: Distance between two hyphen is of 1 cm.

Radiographic investigations to assess limb development produced a total of 368 X-ray images. The number of dogs for each breed that were radiographed at each timepoint are reported in S2 Table. It was not possible to examine Boxers at 12 weeks and Labrador Retrievers beyond 12 weeks of age. Representative radiographs showing the forelimb and hindlimb skeletal development are reported in Figs 3 and 4.

At 6 weeks of age, i.e., the first time point, some ossification centres were already present in all dogs: the proximal and distal epiphyses of the humerus (HumP and HumD, respectively) and the proximal and distal epiphyses of the radius (RadP and RadD, respectively) in the forelimb, the distal epiphysis of the femur (Fem) and the proximal and distal epiphyses of the tibia (TibP and TibD, respectively) in the hindlimb. The remaining OCs were present in a variable number of dogs; the percentage of dogs for each breed in which the OCs were observed at each time point is reported in S3 Table.

At 12 weeks of age, 100% of the dogs presented all the examined OCs, except for the fabellae and popliteus. The former was observed only in all Saarloos Wolfdogs at 12 weeks of age, in 80% of Boxers and White Swiss Shepherd dogs at 14 weeks and in all the observed dogs at 16 weeks (data on Labrador Retrievers are not available); the latter was observed for the first time at 16 weeks of age in only one out of two Saarloos Wolfdogs.

The “OC score” for each breed at each time point is reported in Table 1.

**Table 1:** Breed OC score at each timepoint calculated by summing the number of OCs present out of 14 (HumP, HumD, HumE, RadP, RadD, UlnO, UlnD, Car, Fem, Pat, TibP, TibT, TibD, Tar).

*BREED*	AGE					
	6 w	8 w	10 w	12 w	14 w	16 w
*BOX*	8.0 (8)	12.0 (8)	14.0 (10)	14.0 (6)	14.0 (8)	14.0 (8)
*GS*	11.0 (7)	14.0 (6)	14.0 (7)	14.0 (7)	14.0 (7)	14.0 (7)
*LR*	7.0 (15)	7.0 (11)	13.0 (12)	14.0 (7)	14.0 (7)	14.0 (7)
*SW*	14.0 (9)	14.0 (12)	14.0 (12)	14.0 (12)	14.0 (12)	14.0 (12)
*WSS*	8.0 (4)	14.0 (9)	14.0 (10)	14.0 (10)	14.0 (10)	14.0 (10)

BOX = Boxer; GS = German Shepherd; LR = Labrador Retriever; SW = Saarloos Wolfdog; WSS = White Swiss Shepherd Dog.

Median OC score and number of subjects (in brackets).

The number of OCs present in the investigated breeds was significantly different at 6 weeks of age (p<0.001) and 8 weeks of age (p = 0.006). In particular, after Bonferroni correction, at 6 weeks of age there were significant differences between Boxers and German Shepherds (p = 0.01), Boxers and Saarloos Wolfdogs (p = 0.003), Labrador Retrievers and German Shepherds (p<0.001), Labrador Retrievers and Saarloos Wolfdogs (p<0.001), Labrador Retrievers and White Swiss Shepherd dogs (p = 0.031), Saarloos Wolfdogs and White Swiss Shepherd dogs (p = 0.034). At 8 weeks of age, significant differences were observed between Boxers and Saarloos Wolfdogs (p = 0.006), Boxers and White Swiss Shepherd dogs (p = 0.023), Labrador Retrievers and German Shepherds (p = 0.019), Labrador Retrievers and Saarloos Wolfdogs (p<0.001), Labrador Retrievers and White Swiss Shepherd dogs (p = 0.002).

Radiographic measurements of diaphyseal lengths and OC areas were also performed. Mean and standard deviation of the radiographic measurements, presented by breed, are reported in S4 Table. Graphs illustrating the relative increase in these measures per each breed are reported in S1–S3 Figs.

The differences in the absolute increase of both the diaphyseal length of humerus, radius, ulna and tibia and the areas of the measured OCs were significant during the entire observation period in all the investigated breeds, with greater significance between 6 to 8 and 8 to 10 weeks of age. Non-significant results were found when only three dogs per breed were available and for those OCs (UlnO, Pat, Fib, TibT) whose appearance was delayed in certain breeds (Boxer, Labrador Retriever) (S5 Table).

Regarding breed comparisons, the relative increase in several OC areas and diaphyseal lengths was significantly different between the investigated breeds at various time points (S6 Table).

As for correlations, the OC score was positively correlated with age (R = 0.62; p<0.0001; n = 270) and weight (R = 0.79; p<0.0001; n = 175). The relative increase in the measured OC areas and diaphyseal lengths was negatively correlated with the increasing age and body weight. Higher correlation coefficients were observed with age compared to weight. In particular, a strong correlation between the relative increase of the radiographic measurements and both age and weight was found for: area of the olecranon tuber (aUlnO), area of the patella (aPat), area of the proximal epiphysis of the fibula (aFib), area of the tibial tuberosity (aTibT) and area of the calcaneal tuber (aTar). Moderate but still significant correlations were observed between the dog’s age and the increase in length of the long limb bones (Table 2).

**Table 2:** Spearman’s rank correlation between the relative increase in the radiographic measurements and age and body weight.

	aSca	aHumP	aUlnO	aRadD	lHum	lRad	lUln	lTib	aPat	aFab	aPop	aFib	aTibT	aTar
**AGE**	-.669*** (41)	-.632*** (72)	-.853*** (66)	-.369** (71)	-.523*** (106)	-.479*** (119)	-.508**** (122)	-.683*** (119)	-.942*** (61)	-.563* (14)	.(0)	-.892*** (15)	-.813*** (66)	-.796*** (84)
**WEIGHT**	-.513** (41)	-.334** (72)	-.835*** (66)	-.011 (71)	-.306** (106)	-.252** (119)	-.224* (122)	-.417*** (119)	-.800*** (61)	-.420 (14)	.(0)	-.961*** (15)	-.749*** (66)	-.787*** (84)

*p< 0.05

**p< 0.01

***p< 0.001

****p< 0.0001.

Abbreviations: **aSca** = area of the supraglenoid tubercule; **aHumP** = area of the proximal epiphysis of the humerus; **aUlnO** = area of the olecranon tuber; **aRadD** = area of the distal epiphysis of the radius; **lHum** = diaphyseal length of the humerus; **lRad** = diaphyseal length of the radius; **lUln** = diaphyseal length of the ulna; **lTib** = diaphyseal length of the tibia; **aPat** = area of the patella; **aFab** = area of the fabellae; **aPop** = area of the popliteal bones; **aFib** = area of the proximal epiphysis of the fibula; **aTibT** = area of the tibial tuberosity; **aTar** = area of the calcaneal tuber.

For area measurements, the increase was calculated on the square root. For each parameter correlation coefficient, significance and number of measurements (in brackets) are reported.

The intra-observer ICCs resulted in excellent agreement for all the measurements at all the analysed time points, with values always above 0.98 except for the area of the proximal epiphysis of the fibula (aFib) measurement at 8 weeks of age (0.89). The inter-observer ICCs revealed excellent agreement (>0.92) for all the measurements at all the analysed time points, except for the area of the supraglenoid tubercule (aSca), the distal epiphysis of the radius (aRadD) and the proximal epiphysis of the fibula (aFib) measurements at 8 weeks of age, the area of the supraglenoid tubercule (aSca), the olecranon tuber (aUlnO) and the calcaneal tuber (aTar) measurements at 14 weeks of age, which showed good agreement.

The Bland-Altman plots confirmed a better agreement between intra-observer measurements compared to inter-observer measurements. OC area measurements showed greater intra- and inter-observer variability compared to diaphyseal length measurements. In general, the intra- and inter-observer variability diminished as the dog’s age increased, particularly for area measurements.

## Discussion

Despite the extreme and unique morphological variability of the canine species, the time windows for the appearance of the limb OCs reported in veterinary textbooks are considered universally valid for all dogs, although based on dated studies performed on very small samples of dogs, mainly German Shepherds, Greyhounds and Beagles [4–7,10,11,13,14]. Moreover, the width of these time ranges, which in most cases extends to 4–8 weeks, makes their usefulness rather limited, especially for age estimation purposes. Other studies showed that even breeds of the same size and weight can differ significantly in their growth patterns, therefore putting into question the transferability of data from one breed to another and highlighting the need of further growth studies on a larger number of dogs [1,29,30]. For these reasons, in selecting the breeds to be investigated in this study, an attempt was made to include dogs with different morphotypes, i.e., Lupoid (Saarloos Wolfdog, German Shepherd, White Swiss Shepherd Dog), Molossoid (Boxer) and Braccoid (Labrador Retriever) [31]. The observation timeframe was set between 6 and 16 weeks of age as that is when most of the limb secondary OCs appear. Furthermore, this age interval is crucial from a medico-legal point of view, as 8 weeks is the minimum age at which dogs can be sold and transported in many countries and 15 weeks is the age at which they can be transported internationally within and across the European borders (Regulation EU No 576/2013). Indeed, since age estimation of dogs by dental examination is affected by a high degree of uncertainty [32], the radiographic evaluation of the limb OCs can be used as an alternative or complementary technique in a forensic scenario.

Skeletal growth is essentially determined by genetic factors, but it is also influenced by various external factors, such as nutrition, mechanical loading and disease agents [33]. For example, intermittent mechanical load within the limits of physiological stress stimulates the growth plates, while continuous overloading results in the inhibition of endochondral longitudinal growth [34]. Nutritional deficiencies such as low energy and protein intake, mineral and vitamin deficiencies (in particular, calcium, phosphorus and vitamin D) can determine skeletal deformities, growth retardation and ultimately cessation and weight loss [35]. A recent study aimed at developing evidence-based growth standards for healthy dogs showed that the growth patterns among larger dog breeds are quite diverse and difficult to unify under a common standard curve [36]. Although all the external factors that may influence skeletal growth could not be controlled in our study, all the animals included were in good health conditions on the basis of clinical examination, they were fed with a balanced diet specific for growing dogs and they all had access to appropriate physical exercise; therefore, we suppose that the effect of these factors on our results was minimal.

Our findings showed that the time of appearance of the limb OCs was significantly different even between breeds of the same size. The most precocious breed appeared to be the Saarloos Wolfdog, while German Shepherds and White Swiss Shepherd Dogs followed a similar trend. On the other hand, the Labrador Retriever was the later one. Moreover, in Boxers and Labradors various OCs (i.e., HumE, UlnO, UlnD in the forelimb and Fib, Tar in the hindlimb) showed a delayed appearance compared to the data reported in the literature [16–25]. All the investigated breeds aligned to each other at 12 weeks, although the appearance of the sesamoid bones fabellae and popliteus, which were observed at later stages in a few dogs, might represent another distinguishing element, worthy of further studies.

Morphometry is poorly investigated in the canine species, but it has found application in radiology and ultrasonography to determine the stage of canine pregnancy and to predict the parturition date [37]. Schulze and coll. investigated the postnatal growth of the thoracic and the pelvic limb in four different dog breeds (Great Dane, Rottweiler, Bernese Mountain Dog and Beagle) from birth to maturity, with a measuring tape and a calliper, and found significant differences in the growth patterns of breeds having the same size and weight [29,30]. Regarding radiographic morphometry, one study, dating back to 1982, included only four 13-month-old and four 21-month-old Beagles and radiographs were performed on the dissected femur. The total width of the femur increased significantly with age and body weight, but neither did its length nor its combined cortical thickness [38]. Another morphometric study was recently carried out on 27 spontaneously dead small-sized new-born dogs. Radiographic measurements of the length of long limb bones were positively correlated with the dogs’ age and body weight [39]. However, since this study was carried out on dead animals, it is not possible to make any considerations about bone growth rate in relation to age or body weight.

In the present study, both the diaphyseal length of humerus, radius, ulna and tibia and the areas of the measured OCs increased significantly during the entire observation period in all the investigated breeds, particularly between 6 to 8 and 8 to 10 weeks of age. Non-significant results were only found when three dogs per breed were available, therefore they could be affected by the limited number of measurements. Nevertheless, these findings suggest that limb bone development occurs mainly in the first months of the dog’s life, with decreasing intensity as the animal grows older. This decreasing trend is confirmed by the negative correlation that was found between the increase in radiographic measurements and the dogs’ age and weight.

Breed differences were also observed in the relative increase of the measured OC areas and diaphyseal lengths and were not limited to dogs of different morphotypes. In overall terms, statistically significant differences were most frequently observed between Saarloos Wolfdogs and the other breeds. The OCs that showed a greater variability in their development between the investigated breeds were the olecranon tuber (aUlnO), the patella (aPat) and the tibial tuberosity (aTibT). Breed differences in the relative increase of the area of the proximal epiphysis of the humerus (aHumP) were significant only up to 10 weeks of age. No significant differences were observed in the relative increase of the area of the distal epiphysis of the radius (RadD). When the number of measurements was sufficiently high, significant breed differences could also be observed in the development of the area of the supraglenoid tubercule (aSca), the fabellae (aFab) and the proximal epiphysis of the fibula (aFib). Lastly, breed differences were observed in the relative increase in the diaphyseal length of all the measured long limb bones and, despite the small number of dogs available over 10 weeks of age, it was still possible to observe statistically significant differences even between breeds of the same morphotype, namely Saarloos Wolfdogs and White Swiss Shepherd Dogs.

Since much greater numbers would have been necessary in order to highlight breed-specific differences, correlations were investigated considering the sample population as a whole. A strong positive relationship was found between the number of OCs present and body weight and a moderate but still significant relationship between the number of OCs present and age. These findings suggest that OC appearance might be more influenced by body weight rather than merely by the dog’s age. Stronger correlations between age, weight and the relative increase of the measured OCs were found for the area of the olecranon tuber (aUlnO), the patella (aPat), the proximal epiphysis of the fibula (aFib), the tibial tuberosity (aTibT) and the calcaneal tuber (aTar). These OCs appeared after the 6^th^ week of age in many dogs and showed a more pronounced growth compared to that of the others within the observation window. Remarkably, the growth of the investigated OCs appeared to be more correlated to the dog’s age rather than to its body weight, in contrast to their appearance.

The intra- and inter- observer ICCs resulted in excellent agreement for all the measurements at all the analysed time points, with few exceptions for the inter- observer agreement on some OC area measurements, which was nonetheless good. OC area measurements showed greater intra- and inter-observer variability compared to diaphyseal length measurements; this is most likely due to the fact that it is more difficult to repeat a measurement based on freehand drawing rather than on defined reference points, as in the case of diaphyseal lengths. The intra- and inter-observer variability, particularly for area measurements, diminished as the dog’s age and, accordingly, the dimension and the bone radiopacity of the structure to be measured increased.

Due to the strict legal rules and ethical boundaries concerning the use of dogs for research purposes, this study had to deal with some limitations. For ethical reasons related to the use of privately-owned dogs, it was not conceivable to radiograph the dogs more often than every two weeks. We cannot exclude that this time interval prevented us from detecting further breed differences in the time of appearance of the investigated OCs. It was not always possible to examine a sufficient number of animals in the established schedule due to organizational difficulties and the possibility for the breeders to sell the dogs from 8 weeks of age onwards. Furthermore, in order to reduce the stress for the animals and the exposure to X-rays, the radiographic examinations were performed on-field using a portable X-ray unit and CR system and only one out of the two canonical orthogonal projections required for the radiographic study of the appendicular skeleton was performed. Nevertheless, the equipment used and the machine setting according to Sante’s rule allowed us to obtain radiograms of good quality; the portable X-ray unit, equipped with a 1.2 mm focal spot, provided a good balance between resolution and exposure time. Indeed, since all the procedures in our study were conducted on awake puppies, the speed of acquisition was a limiting factor that needed to be taken care of. The two CR systems employed in this study had similar technical features and therefore they were comparable to each other. Another technical limit is represented by a lack of an internal calibration tool placed closely to the bone structures prior the acquisition of the radiographs. However, the small thickness of the limb bones on one hand, and the precautions we adopted on the other, i.e., maintaining a constant focal distance (100 cm), centring the structures of interest, the direct contact of the limbs to the plate, allowed to satisfactorily reduce geometric distortion and magnification errors. The medio-lateral view of the limbs allowed to visualize and measure a good number of OCs; the manual constraint of the dogs, which required two operators, was well tolerated by all puppies without using pharmacological sedation and allowed us to obtain satisfactory radiograms on the first attempt in most cases. However, maintaining the correct positioning of non-sedated puppies was not always feasible, depending on the temperament of the subjects, and the possibility of repeating the x-ray was bound to the breeder’s consent and availability. The supraglenoid tubercule (Sca) and the proximal epiphysis of the fibula (Fib) were the OCs most affected by mispositioning resulting in the superimposition of thoracic structures or by even a slight rotation of the limb and therefore the most difficult to analyse.

Finally, biological phenomena, such as skeletal development, are affected by variability across and within species, breeds and individuals due to several factors (genetic, epigenetic, nutritional factors). In this study, all the dogs were in good clinical conditions and were raised in similar environments. However, data on puppy locomotion were not collected, so it was not possible to determine the possible effect of mechanical factors on skeletal development.

## Conclusions

This study allowed us to acquire detailed and up-to-date radiographic information regarding limb bone development in skeletally-immature large-sized dogs from 6 to 16 weeks of age. Our preliminary data strongly suggest that differences in limb development exist in dogs of different morphotypes. More specifically, the appearance of the limb secondary OCs occurred earlier in the Saarloos Wolfdog, while the Labrador Retriever seemed to be the later breed. Moreover, in Boxers and Labrador Retrievers various OCs showed a delayed appearance compared to the data reported in the literature. The number of OCs was strongly and positively correlated to body weight. Breed differences were also observed in the relative increase of the measured OC areas and diaphyseal lengths and were not limited to dogs of different morphotypes. In general, statistically significant differences were most frequently observed between Saarloos Wolfdogs and the other breeds. The development of some OCs showed a greater variability between breeds compared to the others and their increase was more strongly correlated with the dog’s age and body weight.

The radiographic examination remains a powerful technique for assessing limb bone development and, thanks to the currently available technologies, it is easily applicable to on-field conditions. The morphometric approach, still poorly investigated in the canine species, proved to be a valuable tool for assessing growth and it is worthy of use in further investigations, also in a medico-legal perspective, in order to better explore its validity in this field and to provide reliable and up-to-date data on a larger number of subjects.

## Supporting information

S1 Fig Relative increase in the measured OC areas in the forelimb of the investigated breeds. A) Area of the supraglenoid tubercule; B) Area of the proximal epiphysis of the humerus; C) Area of the olecranon tuber; D) Area of the distal epiphysis of the radius Colour legend: Black = Boxer (BOX), Blue = German Shepherd (GS), Green = Labrador Retriever (LR), Red = Saarloos Wolfdog (SW), Yellow = White Swiss Shepherd Dog (WSS).
(TIF)

S2 Fig Relative increase in the measured diaphyseal lengths in the forelimb of the investigated breeds. A) Diaphyseal length of the humerus; B) Diaphyseal length of the radius; C) Diaphyseal length of the ulna Colour legend: Black = Boxer (BOX), Blue = German Shepherd (GS), Green = Labrador Retriever (LR), Red = Saarloos Wolfdog (SW), Yellow = White Swiss Shepherd Dog (WSS).
(TIF)

S3 Fig Relative increase in the measured OC areas and diaphyseal lengths in the hindlimb of the investigated breeds. A) Area of the patella; B) Area of the fabellae; C) Area of the proximal epiphysis of the fibula; D) Area of the tibial tuberosity; E) Area of the calcaneal tuber; F) Diaphyseal length of the tibia Colour legend: Black = Boxer (BOX), Blue = German Shepherd (GS), Green = Labrador Retriever (LR), Red = Saarloos Wolfdog (SW), Yellow = White Swiss Shepherd Dog (WSS).
(TIF)

S1 Table Mean weight and standard deviation (Kg) for each breed. Number of subjects reported in brackets.
(PDF)

S2 Table Number of dogs radiographed at each timepoint. (PDF)

S3 Table Percentage of dogs of each breed in which the OCs were present at each time point. HumP, HumD, RadP, RadD, Fem, TibP and TibD were already present in all dogs at 6 weeks of age.
(PDF)

S4 Table Mean and standard deviation of the radiographic measurements, presented by breed. OC areas (a) in mm^2^, diaphyseal lengths (l) in mm. The number of dogs that presented the OC, and in which the OC area/diaphyseal length was measurable, is indicated in brackets.
(PDF)

S5 Table Differences in the absolute increase of the measured OC areas and diaphyseal lengths between the various time points in the investigated breeds (one-tailed Wilcoxon signed rank test). Number of measurements reported in brackets. Three was the minimum number of measurements to perform the test.
(PDF)

S6 Table Differences in the relative increase of the measured OC areas and diaphyseal lengths between the investigated breeds (Wilcoxon rank-sum test). Number of measurements reported in brackets. Three was the minimum number of measurements per breed to perform the test.
(PDF)

S1 Text List of abbreviations. (PDF)
