# Supplementary figures and images for: Limb development in skeletally-immature large-sized dogs: A radiographic study
Source: PLoS One. 2021 Jul 23;16(7):e0254788. doi: 10.1371/journal.pone.0254788 (PMC8301671; doi:10.1371/journal.pone.0254788)

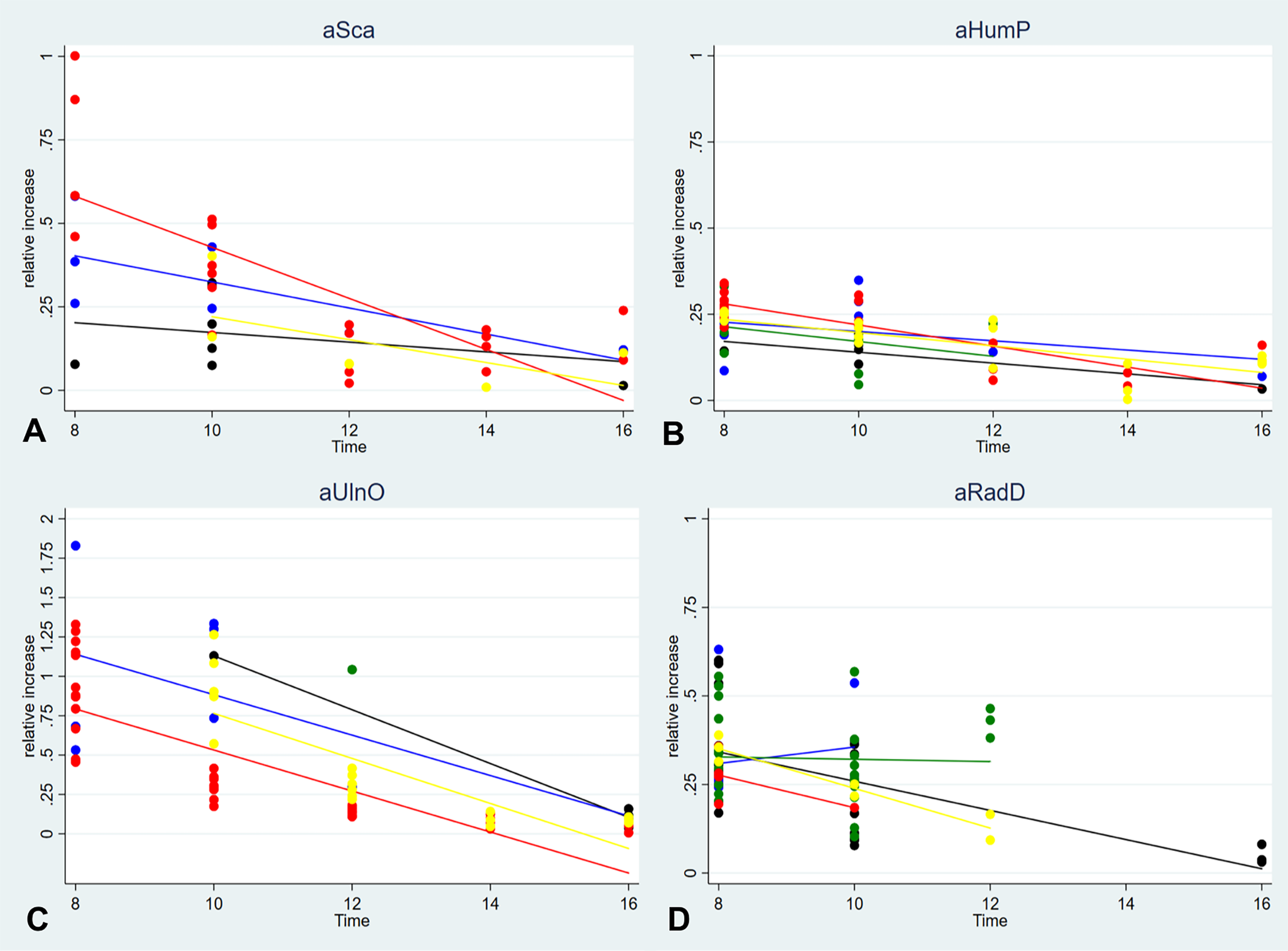

Supplement: S1 Fig — A) Area of the supraglenoid tubercule; B) Area of the proximal epiphysis of the humerus; C) Area of the olecranon tuber; D) Area of the distal epiphysis of the radius Colour legend: Black = Boxer (BOX), Blue = German Shepherd (GS), Green = Labrador Retriever (LR), Red = Saarloos Wolfdog (SW), Yellow = White Swiss Shepherd Dog (WSS). (TIF) [file pone.0254788.s001.tif]

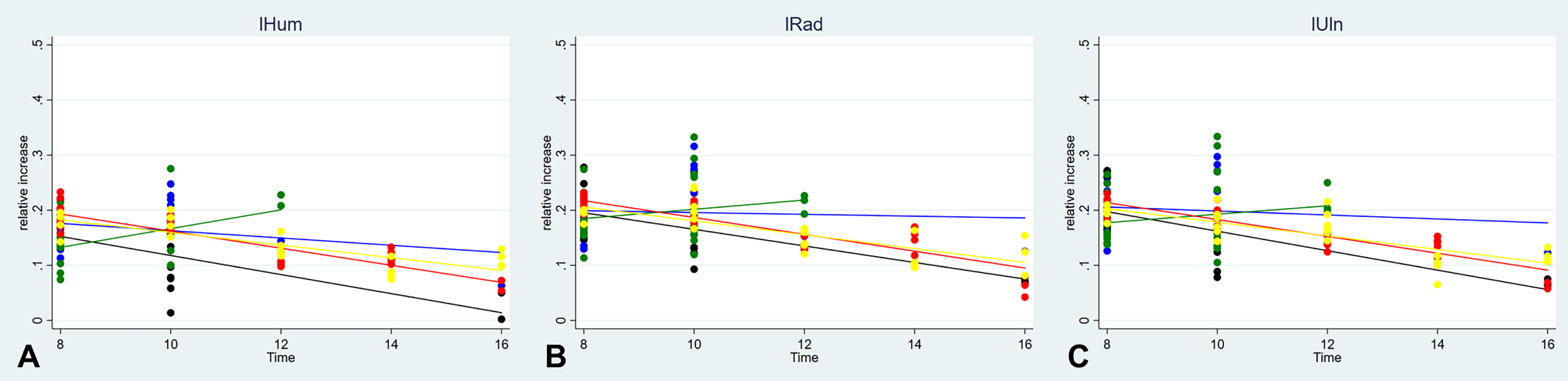

Supplement: S2 Fig — A) Diaphyseal length of the humerus; B) Diaphyseal length of the radius; C) Diaphyseal length of the ulna Colour legend: Black = Boxer (BOX), Blue = German Shepherd (GS), Green = Labrador Retriever (LR), Red = Saarloos Wolfdog (SW), Yellow = White Swiss Shepherd Dog (WSS). (TIF) [file pone.0254788.s002.tif]

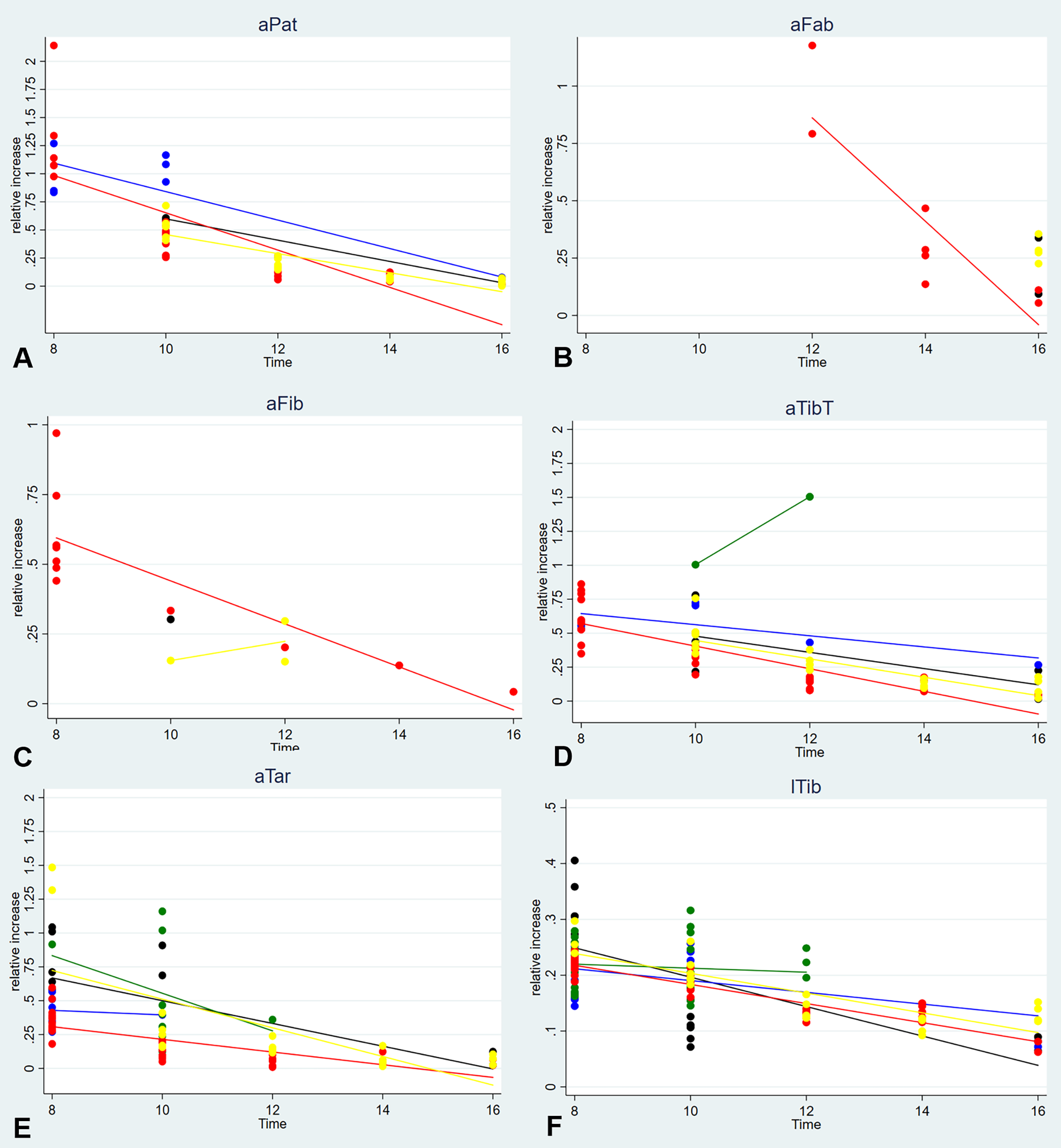

Supplement: S3 Fig — A) Area of the patella; B) Area of the fabellae; C) Area of the proximal epiphysis of the fibula; D) Area of the tibial tuberosity; E) Area of the calcaneal tuber; F) Diaphyseal length of the tibia Colour legend: Black = Boxer (BOX), Blue = German Shepherd (GS), Green = Labrador Retriever (LR), Red = Saarloos Wolfdog (SW), Yellow = White Swiss Shepherd Dog (WSS). (TIF) [file pone.0254788.s003.tif]
