# Supplementary material for: Limb development in skeletally-immature large-sized dogs: A radiographic study
Source: PLoS One. 2021 Jul 23;16(7):e0254788. doi: 10.1371/journal.pone.0254788 (PMC8301671; doi:10.1371/journal.pone.0254788)
Supplement: S1 Table — Number of subjects reported in brackets. (PDF) [file pone.0254788.s004.pdf]

S1 Table. Mean weight and standard deviation (Kg) for each breed. *Number of subjects reported in brackets.*

BREED	AGE (weeks)					
	6	8	10	12	14	16
**BOX**	3.14±0.60 (10)	4.41±0.93 (10)	6.07±1.43 (8)	- (0)	7.96±1.89 (5)	9.65±2.43 (4)
**GS**	3.57±1.03 (7)	5.45±2.28 (7)	7.00±0.93 (4)	10.45±1.34 (2)	12.80 (1)	15.00 (1)
**LR**	2.35±1.24 (15)	3.78±2.04 (15)	4.71±2.47 (14)	8.65±3.69 (7)	- (0)	- (0)
**SW**	3.80±0.52 (12)	5.50±0.61 (12)	7.34±0.70 (8)	9.91±0.75 (6)	12.64±1.51 (6)	14.25±1.77 (2)
**WSS**	3.47±0.34 (10)	5.47±0.56 (10)	7.65±0.81 (6)	9.78±1.76 (6)	11.17±1.28 (5)	14.11±1.46 (4)

Abbreviations: BOX= Boxer; GS= German Shepherd; LR= Labrador Retriever; SW= Saarloos Wolfdog; WSS= White Swiss Shepherd Dog
