# Supplementary material for: Limb development in skeletally-immature large-sized dogs: A radiographic study
Source: PLoS One. 2021 Jul 23;16(7):e0254788. doi: 10.1371/journal.pone.0254788 (PMC8301671; doi:10.1371/journal.pone.0254788)
Supplement: S2 Table — (PDF) [file pone.0254788.s005.pdf]

S2 Table. Number of dogs radiographed at each timepoint.

Breed	Total no. of dogs	No. of dogs examined at:					
		**6 w**	**8 w**	**10 w**	**12 w**	**14 w**	**16 w**
Boxer (BOX)	10	10	10	8	0	5	3
German Shepherd (GS)	7	7	7	4	1	1	1
Labrador Retriever (LR)	15	15	15	14	3	0	0
Saarloos Wolfdog (SW)	12	12	12	8	6	5	2
White Swiss Shepherd Dog (WSS)	10	4	10	6	6	5	4
TOTAL	**54**	**48**	**54**	**40**	**16**	**16**	**10**
PERCENTAGE	**100%**	**89%**	**100%**	**74%**	**29%**	**29%**	**18%**
