# Supplementary material for: Limb development in skeletally-immature large-sized dogs: A radiographic study
Source: PLoS One. 2021 Jul 23;16(7):e0254788. doi: 10.1371/journal.pone.0254788 (PMC8301671; doi:10.1371/journal.pone.0254788)
Supplement: S3 Table — HumP, HumD, RadP, RadD, Fem, TibP and TibD were already present in all dogs at 6 weeks of age. (PDF) [file pone.0254788.s006.pdf]

***S3 Table.* Percentage of dogs of each breed in which the OCs were present at each time point.** HumP, HumD, RadP, RadD, Fem, TibP and TibD were already present in all dogs at 6 weeks of age.

	AGE																													
	6 weeks					8 weeks					10 weeks					12 weeks					14 weeks					16 weeks				
OC	B	G	L	S	W	B	G	L	S	W	B	G	L	S	W	B	G	L	S	W	B	G	L	S	W	B	G	L	S	W
Sca	37.5	100	11.1	100	-	100	100	12.5	100	100	100	100	100	100	100	100	100	100	100	100	100	100	100	100	100	100	100	100	100	100
HumE	0	100	0	100	0	70	100	14.3	100	100	100	100	76.9	100	100	100	100	100	100	100	100	100	100	100	100	100	100	100	100	100
UlnO	0	42.9	0	100	0	30	85.7	6.7	100	100	60	100	64.3	100	100	100	100	100	100	100	100	100	100	100	100	100	100	100	100	100
UlnD	44.4	100	0	100	50	88.9	100	35.7	100	100	100	100	86.7	100	100	100	100	100	100	100	100	100	100	100	100	100	100	100	100	100
Car	0	71.4	0	100	0	70	100	7.1	100	100	100	100	71.4	100	100	100	100	100	100	100	100	100	100	100	100	100	100	100	100	100
Pat	0	42.9	0	66.7	0	44.4	85.7	15.4	100	100	100	100	42.9	100	100	100	100	100	100	100	100	100	100	100	100	100	100	100	100	100
Fab	0	0	0	0	0	0	0	0	0	0	0	0	0	37.5	0	0	0	0	100	0	80	0	-	100	80	100	100	-	100	100
Pop	0	0	0	0	0	0	0	0	0	0	0	0	0	0	0	0	0	0	0	0	0	0	-	0	0	0	0	-	50	0
Fib	0	40	0	100	0	11.1	85.7	10	100	100	57.1	100	66.7	100	100	100	100	100	100	100	100	100	100	100	100	100	100	100	100	100
TibT	0	28.6	0	91.7	0	60	71.4	6.7	100	100	100	100	53.8	100	100	100	100	100	100	100	100	100	100	100	100	100	100	100	100	100
Tar	60	100	6.7	100	33.3	90	100	60	100	100	100	100	93.3	100	100	100	100	100	100	100	100	100	100	100	100	100	100	100	100	100

*Abbreviations: **B**= Boxer; **G**= German Shepherd; **L**= Labrador Retriever; **S**= Saarloos Wolfdog; **W**= White Swiss Shepherd Dog.*

***Sca**= supraglenoid tubercule; **HumP**= proximal epiphysis of the humerus; **HumE**= epiphysis of medial epicondyle of the humerus; **HumD**= distal epiphysis of the humerus; **RadP**= proximal epiphysis of the radius; **RadD**= distal epiphysis of the radius; **UlnO**= olecranon tuber; **UlnD**= distal epiphysis of the ulna; **Car**= accessory carpal bone; **Fem**= distal epiphysis of the femur; **Pat**= patella; **Fab**= Fabellae; **Pop**=Popliteal bones; **Fib**= proximal epiphysis of the fibula; **TibP**= condyles of the proximal epiphysis of the tibia; **TibT**= tibial tuberosity; **TibD**= distal epiphysis of the tibia; **Tar**= calcaneal tuber.*
