# Supplementary material for: Limb development in skeletally-immature large-sized dogs: A radiographic study
Source: PLoS One. 2021 Jul 23;16(7):e0254788. doi: 10.1371/journal.pone.0254788 (PMC8301671; doi:10.1371/journal.pone.0254788)
Supplement: S4 Table — OC areas (a) in mm2, diaphyseal lengths (l) in mm. The number of dogs that presented the OC, and in which the OC area/diaphyseal length was measurable, is indicated in brackets. (PDF) [file pone.0254788.s007.pdf]

S4 Table. Mean and standard deviation of the radiographic measurements, presented by breed.
OC areas (a) in mm², diaphyseal lengths (l) in mm. The number of dogs that presented the OC, and in which the OC area/diaphyseal length was measurable, is indicated in brackets.

4.1 Boxer (BOX)

	AGE					
	**6 w**	**8 w**	**10 w**	**12 w**	**14 w**	**16 w**
aSca	7.40 (1)	10.30±4.09 (8)	16.38±6.43 (5)		37.14±8.47 (5)	34.95±5.73 (2)
aHumP	92.84±11.39 (5)	129.57±18.69 (6)	162.32±20.87 (4)		299.96±55.94 (5)	232.20 (1)
aUlnO	-	6.75±3.46 (2)	20.60±8.59 (4)		57.00±13.16 (5)	68.80±17.35 (3)
aRadD	38.10±12.23 (10)	68.90±14.40 (10)	94.02±16.83 (8)		129.34±19.39 (5)	135.40±32.32 (3)
lHum	63.37±3.18 (6)	72.95±6.49 (10)	79.25±5.33 (6)		98.57±7.78 (4)	102.80±12.28 (3)
lRad	55.46±3.30 (8)	65.13±5.86 (10)	74.55±5.61 (8)		96.38±8.50 (5)	103.5±12.81 (3)
lUln	67.53±5.45 (10)	81.59±7.26 (10)	91.43±7.65 (8)		119.42±9.54 (5)	128.73±14.36 (3)
lTib	57.53±6.60 (9)	73.9±7.15 (9)	81.95±6.98 (8)		108.84±8.93 (5)	117.60±13.85 (3)
aPat	-	17.05±1.81 (4)	28.07±14.57 (7)		92.58±16.38 (5)	97.30±19.02 (3)
aFab	-	-	-		18.17±5.46 (4)	24.83±19.90 (3)
aPop	-	-	-		-	-
aFib	-	7.90 (1)	6.97±5.63 (3)		20.30 (1)	-
aTibT	-	8.56±2.01 (5)	11.79±5.25 (8)		40.340±11.09 (5)	38.60±16.97 (2)
aTar	8.73±2.43 (6)	21.21±9.40 (9)	30.76±7.21 (7)		45.68±9.45 (5)	52.13±14.48 (3)

4.2 German Shepherd (GS)

	AGE					
	**6 w**	**8 w**	**10 w**	**12 w**	**14 w**	**16 w**
aSca	11.02±0.51 (3)	22.17±5.86 (3)	35.25±9.63 (4)	61.29 (1)	61.38 (1)	77.20 (1)
aHumP	158.60±55.33 (4)	182.22±35.50 (5)	277.10±39.02 (4)	385.30 (1)	444.80 (1)	509.10 (1)
aUlnO	18.26±13.74 (3)	35.02±29.99 (5)	49.08±14.86 (4)	98.91 (1)	116.80 (1)	140.80 (1)
aRadD	72.33±23.97 (7)	115.57±52.67 (6)	157.70±25.10 (3)	197.10 (1)	251.20 (1)	-
lHum	64.75±6.13 (7)	74.44±7.37 (7)	85.35±5.06 (4)	103.10 (1)	106.10 (1)	112.80 (1)
lRad	61.01±6.11 (7)	71.34±8.23 (7)	83.95±3.90 (4)	100.30 (1)	107.50 (1)	121.00 (1)
lUln	71.03±5.06 (7)	83.74±8.48 (7)	98.85±4.19 (4)	118.60 (1)	126.50 (1)	142.00 (1)
lTib	65.81±5.41 (7)	78.30±8.66 (7)	90.20±3.96 (4)	-	117.00 (1)	125.40 (1)
aPat	17.55±10.12 (3)	42.87±39.93 (6)	66.51±19.73 (4)	139.50 (1)	161.50 (1)	187.70 (1)
aFab	-	-	-	-	-	38.21 (1)
aPop	-	-	-	-	-	-
aFib	13.90 (1)	14.55±11.87 (2)	17.67±5.36 (3)	-	34.88 (1)	-
aTibT	23.10±2.26 (2)	34.93±23.29 (4)	34.51±12.39 (4)	81.86 (1)	86.08 (1)	138.10 (1)
aTar	22.76±13.92 (6)	51.25±25.29 (5)	74.49±1.94 (2)	-	-	108.90 (1)

*Abbreviations: **aSca**= area of the supraglenoid tubercule; **aHumP**= area of the proximal epiphysis of the humerus; **aUlnO**= area of the olecranon tuber; **aRadD**= area of the distal epiphysis of the radius; **lHum**= diaphyseal length of the humerus; **lRad**= diaphyseal length of the radius; **lUln**= diaphyseal length of the ulna; **lTib**= diaphyseal length of the tibia; **aPat**= area of the patella; **aFab**= area of the fabellae; **aPop**= area of the popliteal bones; **aFib**= area of the proximal epiphysis of the fibula; **aTibT**=area of the tibial tuberosity; **aTar**= area of the calcaneal tuber.*

S4 Table (continued). Mean and standard deviation of the radiographic measurements, presented by breed.
OC areas (a) in mm², diaphyseal lengths (l) in mm. The number of dogs that presented the OC, and in which the OC area/diaphyseal length was measurable, is indicated in brackets.

4.3 Labrador Retriever (LR)

	AGE					
	**6 w**	**8 w**	**10 w**	**12 w**	**14 w**	**16 w**
aSca	-	12.90 (1)	5.15±0.64 (2)	9.60±4.38 (2)		
aHumP	61.25±15.96 (10)	93.98±24.84 (10)	117.61±12.37 (7)	157.70±28.99 (2)		
aUlnO	-	-	15.92±12.77 (9)	26.63±13.57 (3)		
aRadD	25.17±9.84 (12)	48.61±22.68 (15)	78.78±45.28 (14)	83.70±16.53 (3)		
lHum	49.52±6.05 (12)	58.34±8.41 (13)	64.70±9.04 (10)	70.47±6.20 (3)		
lRad	45.45±5.64 (12)	54.67±7.57 (15)	65.01±11.09 (14)	66.73±6.40 (3)		
lUln	57.21±6.64 (13)	68.61±9.05 (15)	80.56±13.85 (14)	83.76±7.35 (3)		
lTib	50.90±6.16 (15)	62.30±8.06 (15)	74.31±11.80 (14)	78.16±7.69 (3)		
aPat	-	-	51.25±60.74 (2)	43.60 (1)		
aFab	-	-	-	-		
aPop	-	-	-	-		
aFib	-	6.90 (1)	6.05±4.44 (4)	-		
aTibT	-	11.70 (1)	22.16±21.19 (7)	16.05±16.33 (2)		
aTar	6.70 (1)	15.14±9.62 (9)	21.55±12.77 (11)	18.67±7.57 (3)		

4.4 Saarloos Wolfdog (SW)

	AGE					
	**6 w**	**8 w**	**10 w**	**12 w**	**14 w**	**16 w**
aSca	6.76±3.78 (4)	20.86±5.59 (11)	37.88±5.82 (6)	44.23±6.48 (6)	55.85±7.68 (4)	74.08±4.22 (2)
aHumP	139.57±18.27 (11)	228.78±26.04 (12)	349.87±40.40 (6)	411.42±24.23 (6)	461.80±28.99 (2)	619.00±35.21 (2)
aUlnO	15.37±7.75 (12)	51.11±9.05 (12)	84.38±7.36 (8)	107.78±6.66 (6)	127.26±3.07 (5)	134.45±3.46 (2)
aRadD	83.56±10.78 (12)	133.02±12.51 (4)	192.05±13.50 (2)	-	200.80 (1)	-
lHum	68.80±2.96 (12)	81.89±2.29 (12)	96.25±2.96 (8)	107.18±3.38 (6)	118.72±2.71 (5)	125.70±2.12 (2)
lRad	64.37±1.59 (12)	77.84±1.86 (12)	93.66±2.56 (8)	107.83±1.92 (6)	123.60±4.09 (5)	130.95±2.33 (2)
lUln	76.35±2.35 (12)	92.30±2.65 (12)	109.95±2.97 (8)	127.15±2.22 (6)	143.72±2.81 (5)	154.45±1.48 (2)
lTib	71.12±1.59 (12)	86.64±1.94 (12)	103.20±2.61 (8)	117.18±1.81 (6)	132.74±3.78 (5)	143.60±1.55 (2)
aPat	14.00±3.99 (6)	55.70±15.56 (12)	108.50±16.78 (8)	134.72±20.04 (6)	153.04±16.23 (5)	162.45±31.32 (2)
aFab	-	-	8.54±0.97 (3)	27.62±7.15 (6)	43.88±7.67 (4)	51.47±10.88 (2)
aPop	-	-	-	-	-	11.41 (1)
aFib	5.84±1.52 (7)	14.83±1.58 (10)	24.81 (1)	31.26±4.12 (3)	39.82±1.11 (2)	40.14±2.91 (2)
aTibT	14.04±5.25 (11)	34.12±6.88 (12)	61.45±9.05 (8)	79.30±7.13 (6)	96.97±6.79 (5)	109.85±6.29 (2)
aTar	30.56±6.43 (12)	55.55±6.96 (12)	73.56±14.51 (8)	77.29±9.78 (6)	82.08±7.66 (5)	92.40±11.88 (2)

Abbreviations: ***aSca****= area of the supraglenoid tubercule;* ***aHumP****= area of the proximal epiphysis of the humerus;* ***aUlnO****= area of the olecranon tuber;* ***aRadD****= area of the distal epiphysis of the radius;* ***lHum****= diaphyseal length of the humerus;* ***lRad****= diaphyseal length of the radius;* ***lUln****= diaphyseal length of the ulna;* ***lTib****= diaphyseal length of the tibia;* ***aPat****= area of the patella;* ***aFab****= area of the fabellae;* ***aPop****= area of the popliteal bones;* ***aFib****= area of the proximal epiphysis of the fibula;* ***aTibT****=area of the tibial tuberosity;* ***aTar****= area of the calcaneal tuber.*

S4 Table (continued). Mean and standard deviation of the radiographic measurements, presented by breed.
OC areas (a) in mm², diaphyseal lengths (l) in mm. The number of dogs that presented the OC, and in which the OC area/diaphyseal length was measurable, is indicated in brackets.

4.5 White Swiss Shepherd Dog (WSS)

	AGE					
	**6 w**	**8 w**	**10 w**	**12 w**	**14 w**	**16 w**
aSca	-	25.47±4.00 (4)	40.68±3.14 (5)	48.52±6.04 (2)	59.67±5.33 (3)	72.98±7.01 (3)
aHumP	122.87±3.96 (3)	194.99±14.78 (9)	272.60±11.29 (5)	380.07±42.77 (3)	423.60±19.35 (5)	518.20±22.25 (4)
aUlnO	-	18.55±7.95 (9)	56.48±8.10 (6)	95.73±7.31 (6)	111.66±8.64 (5)	129.37±8.16 (4)
aRadD	66.47±11.21 (4)	110.63±10.83 (6)	161.28±20.15 (5)	219.70±18.98 (3)	214.90 (1)	-
lHum	62.22±0.53 (4)	73.05±2.38 (10)	84.18±1.85 (5)	95.70±2.23 (6)	104.34±3.43 (5)	114.52±2.78 (4)
lRad	57.17±0.34 (4)	68.47±2.40 (10)	80.92±2.55 (6)	92.52±2.30 (6)	102.96±4.90 (5)	113.27±1.05 (4)
lUln	68.97±.1.08 (4)	82.49±3.23 (10)	95.62±3.35 (6)	112.37±3.70 (6)	123.40±4.57 (5)	136.05±2.94 (4)
lTib	61.15±1.70 (4)	76.25±3.10 (10)	90.0±2.21 (6)	102.30±3.48 (6)	114.04±3.70 (5)	127.87±3.76 (4)
aPat	-	40.97±8.40 (10)	93.45±12.61 (6)	133.48±12.02 (6)	155.40±15.30 (5)	163.97±11.12 (4)
aFab	-	-	-	-	25.54±6.36 (4)	41.80±9.30 (4)
aPop	-	-	-	-	-	-
aFib	-	9.42±7.00 (2)	16.42±4.02 (4)	29.70±6.10 (2)	-	33.40±5.30 (2)
aTibT	-	20.73±5.20 (10)	42.61±3.87 (6)	68.98±6.17 (6)	87.18±7.18 (5)	104.24±4.71 (4)
aTar	6.50±0.28 (4)	37.89±4.37 (10)	58.58±5.23 (5)	77.67±3.36 (6)	87.65±8.78 (5)	95.70±4.54 (4)

*Abbreviations: **aSca**= area of the supraglenoid tubercule; **aHumP**= area of the proximal epiphysis of the humerus; **aUlnO**= area of the olecranon tuber; **aRadD**= area of the distal epiphysis of the radius; **lHum**= diaphyseal length of the humerus; **lRad**= diaphyseal length of the radius; **lUln**= diaphyseal length of the ulna; **lTib**= diaphyseal length of the tibia; **aPat**= area of the patella; **aFab**= area of the fabellae; **aPop**= area of the popliteal bones; **aFib**= area of the proximal epiphysis of the fibula; **aTibT**=area of the tibial tuberosity; **aTar**= area of the calcaneal tuber.*
