# Supplementary material for: Limb development in skeletally-immature large-sized dogs: A radiographic study
Source: PLoS One. 2021 Jul 23;16(7):e0254788. doi: 10.1371/journal.pone.0254788 (PMC8301671; doi:10.1371/journal.pone.0254788)
Supplement: S5 Table — Number of measurements reported in brackets. Three was the minimum number of measurements to perform the test. (PDF) [file pone.0254788.s008.pdf]

S5 Table. Differences in the absolute increase of the measured OC areas and diaphyseal lengths between the various time points in the investigated breeds *(one-tailed Wilcoxon signed rank test). Number of measurements reported in brackets. Three was the minimum number of measurements to perform the test.*

Measure	AGE																								
	8 vs 6 weeks					10 vs 8 weeks					12 vs 10 weeks					14 vs 12 weeks					16 vs 14 weeks				
	BOX	GS	LR	SW	WSS	BOX	GS	LR	SW	WSS	BOX	GS	LR	SW	WSS	BOX	GS	LR	SW	WSS	BOX	GS	LR	SW	WSS
aSca	* (4)	ns (3)	-	* (4)	-	* (5)	-	-	* (6)	-	-	-	-	* (4)	-	-	-	-	* (4)	-	-	-	-	-	-
aHumP	ns (3)	ns (3)	** (7)	** (11)	ns (3)	* (4)	* (4)	* (5)	* (6)	* (5)	-	-	-	* (4)	ns (3)	-	-	-	-	ns (3)	-	-	-	-	* (4)
aUlnO	ns (9)	* (4)	- (14)	** (12)	ns (3)	* (7)	ns (3)	** (12)	** (8)	* (5)	-	-	ns (3)	* (6)	* (6)	-	-	-	* (5)	* (5)	ns (3)	-	-	-	* (4)
aRadD	** (10)	* (6)	** (12)	* (4)	ns (3)	** (8)	ns (3)	*** (14)	-	-	-	-	ns (3)	- (0)	- (2)	-	-	-	-	-	ns (3)	-	-	-	-
lHum	* (3)	** (7)	** (11)	** (12)	* (4)	* (6)	* (4)	** (9)	** (8)	* (5)	-	-	-	* (6)	* (5)	-	-	-	* (5)	* (5)	ns (3)	-	-	-	* (4)
lRad	** (8)	** (7)	** (12)	** (12)	* (4)	** (8)	* (4)	*** (14)	** (8)	* (6)	-	-	ns (3)	* (6)	* (6)	-	-	-	* (5)	* (5)	ns (3)	-	-	-	* (4)
lUln	** (10)	** (7)	** (13)	** (12)	* (4)	** (8)	* (4)	*** (14)	** (8)	* (6)	-	-	ns (3)	* (6)	* (6)	-	-	-	* (5)	* (5)	ns (3)	-	-	-	* (4)
lTib	** (9)	** (7)	*** (15)	** (12)	* (4)	** (7)	* (4)	*** (14)	** (8)	* (6)	-	-	ns (3)	* (6)	* (5)	-	-	-	* (5)	* (4)	ns (3)	-	-	-	* (4)
aPat	* (9)	* (7)	-	** (9)	* (4)	* (7)	* (4)	ns (10)	** (8)	* (6)	-	-	-	* (6)	* (6)	-	-	-	* (5)	* (5)	ns (3)	-	-	-	* (4)
aFab	-	-	-	-	-	-	-	-	* (8)	-	-	-	-	* (6)	-	-	-	-	* (4)	* (5)	ns (3)	-	-	-	* (4)
aPop	-	-	-	-	-	-	-	-	-	-	-	-	-	-	-	-	-	-	-	-	-	-	-	-	-
aFib	ns (9)	-	ns (10)	** (7)	-	* (6)	-	ns (5)	-	-	-	-	-	-	-	-	-	-	-	-	-	-	-	-	-
aTibT	* (10)	* (6)	ns (15)	** (12)	* (4)	** (8)	* (4)	** (13)	** (8)	* (6)	-	-	-	* (6)	* (6)	-	-	-	* (5)	* (5)	-	-	-	-	* (4)
aTar	** (10)	* (5)	** (15)	** (12)	* (4)	** (7)	-	** (12)	** (8)	* (5)	-	-	-	* (6)	* (5)	-	-	-	* (5)	* (5)	ns (3)	-	-	-	* (4)

**p< 0.05; **p< 0.01; ***p< 0.001; "ns"= not significant; "-"= no or insufficient number of measurements*

*Abbreviations: **BOX**= Boxer; **GS**= German Shepherd; **LR**= Labrador Retriever; **SW**= Saarloos Wolfdog; **WSS**= White Swiss Shepherd Dog.*
***aSca**= area of the supraglenoid tubercule; **aHumP**= area of the proximal epiphysis of the humerus; **aUlnO**= area of the olecranon tuber; **aRadD**= area of the distal epiphysis of the radius; **lHum**= diaphyseal length of the humerus; **lRad**= diaphyseal length of the radius; **lUln**= diaphyseal length of the ulna; **lTib**= diaphyseal length of the tibia; **aPat**= area of the patella; **aFab**= area of the fabellae; **aPop**= area of the popliteal bones; **aFib**= area of the proximal epiphysis of the fibula; **aTibT**=area of the tibial tuberosity; **aTar**= area of the calcaneal tuber.*
