# Supplementary material for: Limb development in skeletally-immature large-sized dogs: A radiographic study
Source: PLoS One. 2021 Jul 23;16(7):e0254788. doi: 10.1371/journal.pone.0254788 (PMC8301671; doi:10.1371/journal.pone.0254788)
Supplement: S6 Table — Number of measurements reported in brackets. Three was the minimum number of measurements per breed to perform the test. (PDF) [file pone.0254788.s009.pdf]

S6 Table. Differences in the relative increase of the measured OC areas and diaphyseal lengths between the investigated breeds *(Wilcoxon Rank-Sum Test). Number of measurements reported in brackets. Three was the minimum number of measurements per breed to perform the test.*

Measure	AGE																								
	**8 vs 6 weeks**					**10 vs 8 weeks**					**12 vs 10 weeks**					**14 vs 12 weeks**					**16 vs 14 weeks**				
	**B**	**G**	**L**	**S**	**W**	**B**	**G**	**L**	**S**	**W**	**B**	**G**	**L**	**S**	**W**	**B**	**G**	**L**	**S**	**W**	**B**	**G**	**L**	**S**	**W**
aSca	a (4)	b (3)	a,b,c (5)	c (4)	-	(5)	-	-	(6)	-	-	-	-	-	-	-	-	-	-	-	-	-	-	-	-
aHumP	a,b (3)	c,d (3)	e (7)	a,c,e (11)	b,d (3)	a,b (4)	a,c (4)	c,d (5)	b,d,e (6)	e (5)	-	-	-	(4)	(3)	-	-	-	-	-	-	-	-	-	-
aUlnO	a,b,c (9)	a,d,e (4)	d,f,g (14)	b,f,h (12)	c,e,g,h (3)	(7)	a (3)	(12)	a,b (8)	b (5)	-	-	a,b (3)	a,c (6)	a,b,c (6)	-	-	-	(5)	(5)	(3)	-	-	-	(4)
aRadD	(10)	(6)	(12)	(4)	(3)	(8)	(3)	(14)	-	-	-	-	-	-	-	-	-	-	-	-	-	-	-	-	-
lHum	a (6)	b (7)	a,c (11)	b,c (12)	(4)	a,b,c,d (6)	a,e,f,g (4)	b,e (9)	c,f (8)	d,g (5)	-	-	-	a (6)	a (5)	-	-	-	a (5)	a (5)	a (3)	-	-	-	a (4)
lRad	(8)	a (7)	b (12)	a,b (12)	(4)	a (8)	a,b,c,d (4)	b (14)	c (8)	d (6)	-	-	a,b (3)	a (6)	b (6)	-	-	-	(5)	(5)	a (3)	-	-	-	a (4)
lUln	(10)	(7)	a (13)	a (12)	(4)	a,b,c (8)	a,d,e (4)	(14)	b,d (8)	c,e (6)	-	-	a (3)	a,b (6)	b (6)	-	-	-	a (5)	a (5)	a (3)	-	-	-	a (4)
lTib	a,b,c (9)	a (7)	b (15)	c,e (12)	e (4)	a,b,c (7)	a,d (4)	b (14)	d (8)	c (6)	-	-	a,b (3)	a (6)	b (5)	-	-	-	a (5)	a (4)	a (3)	-	-	-	a (4)
aPat	a,b (9)	c,d (7)	a,c,e,f (11)	e,g (9)	b,d,f,g (4)	a,b (7)	c,d,e (4)	a,c,f,g (10)	b,d,f (8)	e,g (6)	-	-	-	a (6)	a (6)	-	-	-	(5)	(5)	(3)	-	-	-	(4)
aFab	(10)	(7)	(15)	(12)	(4)	(8)	(4)	a (14)	a (8)	(6)	-	-	a (3)	a,b (6)	b (6)	-	-	-	(4)	(5)	(3)	-	-	-	(4)
aPop	(10)	(7)	(15)	(12)	(4)	(8)	(4)	(14)	(8)	(6)	-	-	(3)	(6)	(6)	-	-	-	- (5)	- (5)	(3)	-	-	-	(4)
aFib	a (9)	-	b (10)	a,b (7)	-	(6)	-	(5)	-	-	-	-	-	-	-	-	-	-	-	-	-	-	-	-	-
aTibT	a,b (10)	c,d (6)	a,c,e,f (5)	e,g (12)	b,d,f,g (4)	a (8)	b,c (4)	(13)	a,b (8)	c (6)	-	-	-	a (6)	a (6)	-	-	-	(5)	(5)	-	-	-	-	-
aTar	a,b,c (10)	a,d (5)	(15)	b,e (12)	c,d,e (4)	a (7)	-	b (12)	a,b,c (8)	c (5)	-	-	-	a (6)	a (5)	-	-	-	(5)	(5)	(3)	-	-	-	(4)

Common letters indicate significant pairwise differences (p< 0.05); "-"= no or insufficient number of measurements.

*Abbreviations: **B**= Boxer; **G**= German Shepherd; **L**= Labrador Retriever; **S**= Saarloos Wolfdog; **W**= White Swiss Shepherd Dog.*
***aSca**= area of the supraglenoid tubercule; **aHumP**= area of the proximal epiphysis of the humerus; **aUlnO**= area of the olecranon tuber; **aRadD**= area of the distal epiphysis of the radius; **lHum**= diaphyseal length of the humerus; **lRad**= diaphyseal length of the radius; **lUln**= diaphyseal length of the ulna; **lTib**= diaphyseal length of the tibia; **aPat**= area of the patella; **aFab**= area of the fabellae; **aPop**= area of the popliteal bones; **aFib**= area of the proximal epiphysis of the fibula; **aTibT**=area of the tibial tuberosity; **aTar**= area of the calcaneal tuber.*
