# Supplementary material for: Limb development in skeletally-immature large-sized dogs: A radiographic study
Source: PLoS One. 2021 Jul 23;16(7):e0254788. doi: 10.1371/journal.pone.0254788 (PMC8301671; doi:10.1371/journal.pone.0254788)
Supplement: S1 Text — (PDF) [file pone.0254788.s010.pdf]

**LIST OF ABBREVIATIONS** (in alphabetical order)

**aFab**= area of the fabellae

**aFib**= area of the proximal epiphysis of the fibula

**aHumP**= area of the proximal epiphysis of the humerus

**aPat**= area of the patella

**aPop**= area of the popliteal bones

**aRadD**= area of the distal epiphysis of the radius

**aSca**= area of the supraglenoid tubercule

**aTar**= area of the calcaneal tuber

**aTibT**=area of the tibial tuberosity

**aUlnO**= area of the olecranon tuber

**BOX**= Boxer

**Car**= accessory carpal bone

**Fab**= Fabellae

**Fem**= distal epiphysis of the femur

**Fib**= proximal epiphysis of the fibula

**HumD**= distal epiphysis of the humerus

**HumE**= epiphysis of medial epicondyle of the humerus

**HumP**= proximal epiphysis of the humerus

**GS**= German Shepherd

**lHum**= diaphyseal length of the humerus

**LR**= Labrador Retriever

**lRad**= diaphyseal length of the radius

**lTib**= diaphyseal length of the tibia

**lUln**= diaphyseal length of the ulna

**OC(s)**= ossification centre(s)

**Pat**= patella

**Pop**=Popliteal bones

**RadD**= distal epiphysis of the radius

**RadP**= proximal epiphysis of the radius

**Sca**= supraglenoid tubercule

**SW**= Saarloos Wolfdog

**Tar**= calcaneal tuber

**TibD**= distal epiphysis of the tibia

**TibP**= condyles of the proximal epiphysis of the tibia

**TibT**= tibial tuberosity

**UlnD**= distal epiphysis of the ulna

**UlnO**= olecranon tuber

**WSS**= White Swiss Shepherd Dog
